# Herpes Simplex Virus Type 2 (HSV-2) and Cytomegalovirus (CMV) among Women with Macerated Stillbirth: A Cross-Sectional Hospital-Based Study from Mwanza, Tanzania

**DOI:** 10.1155/2022/2156835

**Published:** 2022-09-08

**Authors:** Helmut A. Nyawale, Elieza Chibwe, Fridolin Mujuni, Lidya Maiga, Albert Silvin, Alda Ester Chongo, Bertrand Msemwa, Vitus Silago, Mtebe Majigo, Doreen Kamori, Stephen E. Mshana, Mariam M. Mirambo

**Affiliations:** ^1^Department of Microbiology and Immunology, Weill Bugando School of Medicine, Catholic University of Health and Allied Sciences, P.O. Box 1464, Mwanza, Tanzania; ^2^Department of Obstetrics and Gynecology, Weill Bugando School of Medicine, Catholic University of Health and Allied Sciences, P.O. Box 1464, Mwanza, Tanzania; ^3^Department of Biological Sciences, Eduardo Mondlane University, Maputo, Mozambique; ^4^Institute of Allied Health Sciences, Catholic University of Health and Allied Sciences, P.O. Box 1464, Mwanza, Tanzania; ^5^Department of Microbiology and Immunology, Muhimbili University of Health and Allied Sciences, P.O. Box 65001, Dar es Salaam, Tanzania

## Abstract

**Background:**

Stillbirth adversely affects pregnancy outcomes in low- and middle-income countries (LMICs). Viral infections have been implicated as one of the causes of stillbirths. Despite high rates of stillbirths and high viral prevalence in LMICs, there is limited information regarding their association. This study investigated the magnitude of herpes simplex 2 virus (HSV-2) and human cytomegalovirus (HCMV) among women with macerated stillbirth.

**Methods:**

A cross-sectional hospital-based study was conducted involving 279 women with macerated stillbirth between July and August 2018 at different health facilities in Mwanza, Tanzania. Detection of HSV-2 was done by immunochromatographic test while that of HCMV was done using enzyme-linked immunosorbent assay (ELISA). Descriptive data analysis was done using STATA version 13.

**Results:**

A total of 28 (10.04%, 95% CI: 6.8-13.9) tested positive for HSV-2 IgG antibodies with only 4 (1.43%, 95% CL: 0.3-2.8) testing positive for HSV-2 IgM antibodies. HCMV IgG antibodies were detected in 131 (77.98%, 95% CI: 71-84) of 168 women tested. By multivariate logistic regulation analysis, advanced age (OR: 0.93, 95% CI: 0.87-0.99, *p* = 0.025) was significantly associated with negative HSV-2 IgG antibodies. By log multinomial regression analysis, only urban residence (RRR.4.43: 95% CI 1.53-12.80, *p* = 0.006) independently predicted HCMV IgG seropositivity among women with stillbirth. Twenty-one (30.9%) of women with positive HCMV IgG antibodies had low avidity index (<40%) indicating recent infection.

**Conclusion:**

Significant proportion of women with macerated stillbirth residing in urban and with low age have HCMV and HSV antibodies, respectively. This calls for the need to consider introducing screening of these infections in the Tanzanian antenatal package and further studies to explore the role of these viruses in causing stillbirth in Tanzania.

## 1. Background

Vertical transmission of cytomegalovirus (CMV) and human simplex viruses (HSV) has been associated with disabling and potentially fatal effects on the fetus. Worldwide, cytomegalovirus (CMV) is regarded as one of the most common congenital infections and can cause hearing loss and neurodevelopmental disorders. Worldwide, it is estimated about 2-3% of pregnant women are infected with HSV [1]. Furthermore, based on the systematic review and meta-analysis, 83% of general population are seropositive for CMV with estimated seroprevalence of 86% being reported in women of childbearing age [2]. Vertical transmission during pregnancy is rare occurring in less than 1% of cases, but for those with active lesions or shedding the virus asymptomatically, the risk of vertical transmission intrapartum is high. Stillbirth is among the most devastating obstetric complications in resource-constrained countries [3]. By 2015, there were 2.6 million cases of stillbirth worldwide with high prevalence reported in low- and middle-income countries (LMICs) [4–6]. In recent years, there has been reduction of stillbirth cases in high-income countries (HICs) due to improved antenatal care (ANC) as compared to LMICs. In most of sub-Saharan African countries including Tanzania, the rates of stillbirths are still high with approximately 47,000 cases annually in Tanzania [7]. Despite high number of cases in most of these countries, causes have not been well studied. In Tanzania, much has been done to reduce stillbirth cases; however, there are infections which are known to contribute to cases of stillbirths elsewhere [8–10]. Maternal infections accounts for up to 25% and 50% of stillbirth cases in HICs and LMICs, respectively [11–13].

Viruses such as rubella, parvovirus B19, herpes simplex 2 virus (HSV-2), and human cytomegalovirus (HCMV) are common in Mwanza, Tanzania [14–17]. Primary human cytomegalovirus (HCMV) infection is associated with transplacental transmission in about 30%-40% of maternal infection [18]. HCMV and HSV-2 have been associated with cases of stillbirth worldwide with high rates reported in LMICs [8, 9]. This study for the first time in Mwanza is aimed at determining seroprevalence of these viruses among women with macerated stillbirth.

## 2. Materials and Methods

### 2.1. Study Design and Study Settings

A cross-sectional hospital-based study was conducted between July and August 2018 in nine health facilities located in rural and urban areas in Mwanza. These health facilities were Bugando Medical Centre, Sekou Toure Regional Referral Hospital, Nyamagana District Hospital, Sengerema Designated District Hospital, Misungwi District Hospital, Ngudu District Hospital, Sumve and Butimba Hospitals, and Magu District Hospital (Table 1).

### 2.2. Study Participants

Study participants were women aged 18 years and above presenting with stillbirths.

### 2.3. Sample Size Estimation and Sampling Technique

The sample size was calculated by Kish Leslie formula using the prevalence of 20.7% [19]. The minimum sample size estimated was 252. Serial sampling of women who met the inclusion criteria was performed until the sample size was reached.

### 2.4. Inclusion Criteria

The study included women aged 18 years and above presenting with stillbirths.

### 2.5. Exclusion Criteria

The women whose cause of stillbirth was known such as antepartum hemorrhage, hypertensive disorder, labor related, and severe anemia were excluded.

### 2.6. Data Collection and Sample Collection from the Participants

Data were collected using a pretested questionnaire. Variables collected included social demographic, maternal, foetal health systems, and ANC and intrapartum characteristics (Table 2). Four-five millilitres of whole blood was collected and transferred to plain vacutainer tubes (Becton, Dickinson, and company, Nairobi, Kenya). Sera was separated and then stored in cryovials at -40°C freezer until processing.

### 2.7. Diagnostic Tests and Laboratory Procedures and Case Definition

Detection of HSV-2 was done using immunochromatographic tests following manufacturer's instructions (Exact Diagnostic Devices, USA), while detection of HCMV IgM and IgG antibodies was done using enzyme-linked immunosorbent assay as per the manufacturer's instructions (PishtazTeb, Tehran, Iran). All IgG samples with high titters were subjected to avidity assay as previously described [20, 21]. In this study, positive IgG and IgM for HSV-2 and HCMV were defined as case, therefore outcome of this study.

### 2.8. Statistical Analysis

Data were cleaned, coded, and analyzed using STATA version 13.0. Percentage/fraction was used to summarize categorical variables, while mean (STD)/median (IQR) was used to summarize continuous variables. *T-*test and Wilcoxon's rank-sum (Mann-Whitney) tests were used to compare means and medians among various groups, respectively. Logistic regression analysis and log multinomial were used to show association between dependent and independent variables for HSV and HCMV seropositivity, respectively. *P* value of < 0.05 at 95% confidence interval was considered as statistically significant.

### 2.9. Ethical Statement

Ethical clearance for conducting this study was sought from CUHAS/BMC Research Ethics and Review Committee (CREC), CREC/613/2018. Permission of doing the study was obtained from relevant government authorities and specific health facilities.

## 3. Results

### 3.1. Sociodemographic Characteristics of the Enrolled Women with Macerated Stillbirth

A total of 279 women with macerated stillbirth were enrolled in this study with median age of 27 and IQR of 22-34 years. More than a half 93(55.36%) of women were from rural areas. Other characteristics are shown in Table 2.

### 3.2. Seropositivity of HSV-2 IgG and IgM Antibodies among Women with Macerated Stillbirth in Mwanza, Tanzania (*n* = 279)

Out of 279 women tested, 28 (10.04%, 95% CI: 6.8-13.9) tested positive for HSV-2 IgG antibodies, while 4 (1.43%, 95% CI: 0.3-2.8) tested positive for HSV-2 IgM antibodies. The median age of IgG seropositive women was significantly lower than that of IgG seronegative women (24.5, IQR: 19.5-29 vs. 27, IQR: 22-34 years, *p* = 0.009).

### 3.3. Factors Associated with the HSV-2 IgG Seropositivity among Women with Macerated Stillbirth in Mwanza Tanzania (*n* = 279)

On univariate analysis, advanced age (OR: 0.92, 95% CI: 0.86-0.98, *p* = 0.015) significantly protected women from being IgG seropositive. Advanced age (OR: 0.93, 95% CI: 0.87-0.99, *p* = 0.025) remained significantly associated with HSV-2 IgG protection on multitvariate logistic regression analysis (Table 3).

### 3.4. Seropositivity of HCMV IgM and IgG Antibodies among Women with Macerated Stillbirth in Mwanza, Tanzania (*n* = 168)

Out of 168, 131 (77.98%, 95% CI: 71-84) tested positive for HCMV IgG antibodies, while none of them was IgM seropositive.

### 3.5. Factors Associated with HCMV IgG Seropositivity among Women with Macerated Stillbirth in Mwanza Region

On univariate log binomial regression analysis, residing in urban areas (RRR: 7.34: 95% CI 2.61-20.03 *p* = 0.000) was significantly associated with HCMV IgG seropositivity and remained significant on log multinomial regression analysis (RRR.4.43: 95% CI 1.53-12.80, *p* = 0.006) (Table 4).

### 3.6. HCMV IgG Avidity Index Values

**Figure 1:**
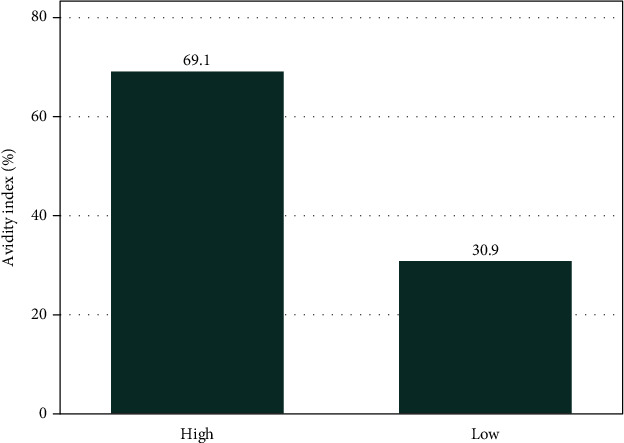
Avidity index values of IgG seropositive individuals with high titters.

Avidity assay of HCMV IgG antibodies positive samples revealed that 21 (30.9%) of the samples had low avidity index indicating recent infections (Figure 1).

## 4. Discussion

About one-tenth of study population tested positive for HSV-2 IgG antibodies with about one percent testing positive for HSV-2 IgM antibodies. Furthermore, it was confirmed that the majority of study participants were positive for HCMV IgG antibodies with about a third having low avidity index (<40%) indicating recent infection. Urban residence independently predicted HCMV IgG seropositivity among women with stillbirth.

This is the first study to investigate maternal immunoreactivity to HSV-2 and HCMV among women with macerated stillbirth in Mwanza, Tanzania. The seropositivity of HSV-2 IgG antibodies was found to be 10.4% comparable to a previous study in Nepal that reported seropositivity of 10% among women with spontaneous abortion [22]. In comparison to previous studies among women with abortions and stillbirth in Iraq and Nigeria, the seropositivity in the current study is significantly low [8, 23]. This could be due to the fact that these previous studies used ELISA assays which are more sensitive than immunochromatographic test. In contrast to a previous study in the same setting, only decrease in age was associated with HSV-2 IgG seropositivity [8]. This could be explained by the fact that as the age increases, antibody titters tend to decrease as reported previously [24, 25].

Regarding HCMV IgG seropositivity, it was found to be high (77.98%) as in previous studies among pregnant women in Mwanza as well as among pregnant women in Sudan [26, 27]. This observation is also similar to a previous study among pregnant women and women with recurrent abortion in Russia [28]. Only residing in urban areas was significantly associated with IgG seropositivity which is similar to previous studies conducted among pregnant women in Mwanza and among women of child bearing age in India [26, 29]. High populated areas have been associated with high HCMV transmission due to poor living conditions [30]. About a third (31%) of women had low avidity index reflecting recent infections signifying high transmission of HCMV in Mwanza and possible cause of poor pregnancy outcomes [9].

## 5. Limitation of the Study

The study was done in Mwanza, Tanzania; therefore, this study may not be representative of the whole country at large. In addition, this study used rapid immunochromatographic tests which have been found to have low sensitivity compared to ELISA assay; therefore, prevalence of HSV-2 IgG and IgM might have been underestimated.

## 6. Conclusion and Recommendations

The seropositivity of HSV-2 IgG antibodies among women with macerated stillbirth and low age in Mwanza is high. In addition, the HCMV infections are significantly high among women with stillbirth residing in urban areas in Mwanza. There is a need of including HSV-2 and HCMV screening services in Tanzania antenatal package.

## Figures and Tables

**Table 1:** Summary of hospitals involved in this study.

SNO	Hospital	Location	Deliveries per month	Stillbirth per month
1	Bugando Medical Centre	Nyamagana	740	10
2	Sekou Toure	Nyamagana	700	20
3	Nyamagana District hospital	Nyamagana	449	15
4	Magu District hospital	Magu	230	9
5	Sumve Hospital	Misungwi	130	3
6	Sengerema Hospital	Sengerema	746	27
7	Misungwi Hospital	Misungwi	200	4
8	Bukumbi Hospital	Misungwi	82	2
9	Ngudu District Hospital	Kwimba	245	7

**Table 2:** Sociodemographic characteristics and obstetrics history of 279 women with macerated stillbirth in Mwanza Tanzania.

Participant's characteristics	Number (*n*)/median/mean	Percentage/IQR/SD
Age	279	27.76 ± 7.22
Gravidity	279	3.25 ± 2.39
Parity	279	2.93 ± 2.18
Gestation age	279	35.62 ± 3.58
Education		
Primary	229	82.08%
Secondary	36	12.90%
Tertiary	14	5.02%
Occupation		
Business	44	15.77%
Employed	25	8.96%
Farming	178	63.80%
Unemployed/employed	32	11.47%
Marital status		
Married	228	81.72%
Single	51	18.28%
Fever		
Yes	73	26.14%
No	206	73.84%
G/rash		
Yes	25	8.96%
No	254	91.04%
Headache		
Yes	87	31.18%
No	192	68.82%
History of abortion		
Yes	51	18.28%
No	228	68.82%
History congenital malformation		
Yes	1	0.36%
No	278	99.64%

**Table 3:** Univariate and multivariate logistic regression analyses of the factors associated with HSV-2 IgG seropositivity among 279 women with stillbirth in Mwanza, Tanzania.

Variables	Mean (SD) (%)	Univariate, OD (95%CL)	*P* value	Multivariate OD (95%CL)	*P* value
Age	27.76 ± 7.22	0.92 (0.86-0.98)	0.015	0.93 (0.87-0.99)	0.025
Residence					
Rural	16 (10.26%)	1.0			
Urban	12 (7.76%)	0.95 (0.43-2.08)	0.890		
Parity	2.93 ± 2.18	0.92 (0.76-1.12)	0.414		
Education					
Primary	24 (10.48%)	1.0			
Secondary	3 (8.33%)	1.18 (1.11-12.42)	0.889		
Tertiary	1 (7.14%)	1.52 (0.19-12.15)	0.692		
Occupation					
Unemployed	4 (12.50%)	1.0			
Business	4 (9.09%)	1.15 (0.19-6.77)	0.877		
Employed	2 (8.0%)	1.64 (0.27-9.78)	0.586		
Farming	18 (10.11%)	1.28 (0.28-5.94)	0.741		
Marital status					
Single	8 (15.69%)	1.0			
Married	20 (8.77%)	1.93 (0.79-4.68)	0.143	1.45 (0.58-3.64)	0.418
Fever					
No	21 (10.19%)	1.0			
Yes	7 (9.59%)	0.93 (0.38-2.29)	0.882		
H/rash					
No	26 (10.24%)	1.0			
Yes	2 (8.00%)	0.76 (0.17-3.42)	0.723		
Headache					
No	21 (10.94%)	1.0			
Yes	7 (8.05%)	0.71 (0.29-1.74)	0.458		
H/abortion					
No	25 (10.96%)	1.0			
Yes	3 (5.88%)	0.51 (0.15-1.75)	0.283		
H/low birth weight					
No	23 (9.66%)	1.0			
Yes	5 (12.20%)	1.29 (0.46-3.63)	0.619		
Stillbirth					
No	21 (11.35%)	1.0			
Yes	7 (7.45%)	0.62 (0.25-1.54)	0.308		
HIV status					
Negative	26 (9.96%)	1.0			
Positive	2 (11.11%)	1.12 (0.25-5.19)	0.8		

**Table 4:** Univariate and multinomial logistic regression analysis of the factors associated with HCMV IgG seropositivity among 168 women with stillbirth in Mwanza, Tanzania.

Variable	HCMV Seropositivity%/mean SD, median IQR	Univariate analysis		Multivariate analysis	
		RRR: 95% CI	*P* value	RRR: 95% CI	*P* value
Age	27, IQR: 22-34	1.02 (0.97-1.07)	0.406		
Parity	2.92 ± 2.2	0.99 (0.85-1.16)	0.926		
Gestation age	37 IQR:33-38	1.00 (0.91-1.10)	0.999		
Residence					
Rural	61 (65.59%)	1			
Urban	70 (93.33%)	7.34 (2.69-20.03)	<0.001	4.43 (1.53-12.80)	0.006
Education level					
None	12 (70.59%)	1			
Primary	89 (76.72%)	0.82	0.992		
Secondary	19 (79.17%)	0.94	0.992		
Tertiary	11 (100%)	1.3	0.992		
Occupation					
Unemployed	18 (90.00%)	1			
Business	76 (70.37%)	0.26 (0.06-1.2)	0.085	0.50 (0.10-2.50)	0.400
Farming	18 (85.71%)	0.39 (0.11-1.44	0.159	0.61 (0.15-2.38)	0.481
S/employed	18 (90.00%)	NA	NA		NA
Marital status					
Single	20 (71.43%)	1			
Married	111 (79.29%)	0.65 (0.26-1.63)	0.362		
History of abortion					
No	106 (79.70%)	1			
Yes	25 (71.43%)	1.57 (0.67-1.66)	0.296		
History of LBW					
No	113 (79.58%)	1			
Yes	18 (69.23%)	1.73 (0.68-4.38)	0.246		
History of premature birth					
No	126 (78.26%)	1			
Yes	5 (71.43%)	1.73 (0.68-4.38)			

## Data Availability

All data generated during this study are included in this manuscript.

## References

[1] Hammad W. A. B., Konje J. C. (2021). Herpes simplex virus infection in pregnancy - an update. *European Journal of Obstetrics & Gynecology and Reproductive Biology*.

[2] Navti O. B., Al-Belushi M., Konje J. C. (2021). Cytomegalovirus infection in pregnancy - an update. *European Journal of Obstetrics & Gynecology and Reproductive Biology*.

[3] Saleem S., Tikmani S. S., McClure E. M. (2018). Trends and determinants of stillbirth in developing countries: results from the global network’s population-based birth registry. *Reproductive Health*.

[4] Lawn J., Blencowe H., Waiswa P. (2016). for The Lancet Ending Preventable Stillbirths Series study group with The Lancet Stillbirth Epidemiology investigator group. Stillbirths: rates, risk factors, and acceleration towards 2030. *Lancet*.

[5] Aminu M., Unkels R., Mdegela M., Utz B., Adaji S., van den Broek N. (2014). Causes of and factors associated with stillbirth in low- and middle-income countries: a systematic literature review. *BJOG: An International Journal of Obstetrics & Gynaecology*.

[6] World Health Organization (2016). *World Health Statistics 2016: Monitoring Health for the SDGs Sustainable Development Goals*.

[7] Karin P., Katarina B., Roger B. (2002). Diagnostic evaluation of intrauterine fetal deaths in Stockholm 1998–99. *Acta Obstetricia et Gynecologica Scandinavica*.

[8] Naqid I. A., Yousif S. H., Hussein N. R. (2020). Seroprevalence of rubella and herpes simplex virus in women with miscarriage and stillbirth in Zakho city, Kurdistan region, Iraq: a cross-sectional study. *Women’s Health Bulletin*.

[9] Iwasenko J. M. H. J., Arbuckle S., Graf N., Hall B., Craig M. E., Rawlinson W. D. (2011). Human cytomegalovirus infection is detected frequently in stillbirths and is associated with fetal thrombotic vasculopathy. *Journal of Infectious Diseases*.

[10] Rawlinson W. D. H. B., Jones C. A., Jeffery H. E. (2008). Viruses and other infections in stillbirth: what is the evidence and what should we be doing?. *Pathology*.

[11] McClure E., Nalubamba-Phiri M., Goldenberg R. (2006). Stillbirth in developing countries. *International Journal of Gynecology & Obstetrics*.

[12] Goldenberg R. L., McClure E. M., Saleem S., Reddy U. M. (2010). Infection-related stillbirths. *The Lancet*.

[13] Goldenberg R. L., Thompson C. (2003). The infectious origins of stillbirth. *American Journal of Obstetrics and Gynecology*.

[14] Mirambo M. M., Aboud S., Majigo M., Groβ U., Mshana S. E. (2019). Adverse pregnancy outcomes among pregnant women with acute Rubella infections in Mwanza city, Tanzania. *International Journal of Infectious Diseases*.

[15] Mirambo M. M., Maliki F., Majigo M. (2017). The magnitude and correlates of parvovirus B19 infection among pregnant women attending antenatal clinics in Mwanza, Tanzania. *BMC Pregnancy and Childbirth*.

[16] Mirambo M., Chibwe E., Mushi M., Majigo M., Mshana S. (2016). Cytomegalovirus, parvovirus B19 and rubella co-infection among pregnant women attending antenatal clinics in Mwanza City: the need to be considered in Tanzanian antenatal care package. *Epidemiology (Sunnyvale)*.

[17] Mirambo M. M., Isdori C., Mshana S. E. (2017). Serological profiles of herpes simplex virus type 2 among HIV negative population in Mwanza City, Tanzania. *Tanzania Journal of Health Research*.

[18] Ross D. S., Dollard S. C., Victor M., Sumartojo E., Cannon M. J. (2006). The epidemiology and prevention of congenital cytomegalovirus infection and disease: activities of the centers for disease control and prevention workgroup. *Journal of Women's Health*.

[19] Yahya-Malima K. I., Evjen-Olsen B., Matee M. I., Fylkesnes K., Haarr L. (2008). HIV-1, HSV-2 and syphilis among pregnant women in a rural area of Tanzania: prevalence and risk factors. *BMC Infectious Diseases*.

[20] Prince H. E., Lapé-Nixon M., Novak-Weekley S. M. (2014). Performance of a cytomegalovirus IgG enzyme immunoassay kit modified to measure avidity. *Clinical and Vaccine Immunology*.

[21] Bodéus M., Feyder S., Goubau P. (1998). Avidity of IgG antibodies distinguishes primary from non-primary cytomegalovirus infection in pregnant women. *Clinical and Diagnostic Virology*.

[22] Acharya D., Shrestha A., Bogati B., Khanal K., Shrestha S., Gyawali P. (2014). Serological screening of TORCH agents as an etiology of spontaneous abortion in Dhulikhel Hospital, Nepal. *American Journal of Biomedical and Life Sciences*.

[23] Kalu E., Ojide C., Chuku A. (2015). Obstetric outcomes of human herpes virus-2 infection among pregnant women in Benin, Nigeria. *Nigerian journal of clinical practice*.

[24] Kamali A., Nunn A., Mulder D., Van Dyck E., Dobbins J., Whitworth J. (1999). Seroprevalence and incidence of genital ulcer infections in a rural Ugandan population. *Sexually Transmitted Infections*.

[25] Weiss H., Buve A., Robinson N. (2001). The epidemiology of HSV-2 infection and its association with HIV infection in four urban African populations. *AIDS*.

[26] Chibwe E., Mirambo M. M., Kihunrwa A., Mshana S. E. (2017). Magnitude of the cytomegalovirus infection among pregnant women attending antenatal clinics in the city of Mwanza, Tanzania. *BMC Research Notes*.

[27] Hamdan H. Z., Abdelbagi I. E., Nasser N. M., Adam I. (2011). Seroprevalence of cytomegalovirus and rubella among pregnant women in western Sudan. *Virology Journal*.

[28] Odland J. Ø., Sergejeva I. V., Ivaneev M. D., Jensen I. P., Stray-Pedersen B. (2001). Seropositivity of cytomegalovirus, parvovirus and rubella in pregnant women and recurrent aborters in Leningrad county, Russia. *Acta obstetricia et gynecologica Scandinavica*.

[29] Jindal N., Aggarwal A., Sheevani (2005). A pilot seroepidemiological study of cytomegalovirus infection in women of child bearing age. *Indian Journal of Medical Microbiology*.

[30] Neirukh T., Qaisi A., Saleh N. (2013). Seroprevalence of cytomegalovirus among pregnant women and hospitalized children in Palestine. *BMC Infectious Diseases*.

